# Phenotypic Shifts in Macrophages Within Advanced Atherosclerotic Plaques in Humans

**DOI:** 10.1096/fba.2025-00037

**Published:** 2025-05-05

**Authors:** Barbora Muffova, Sona Kauerova, Hana Bartuskova, Karel Paukner, Libor Janousek, Helena Cermakova, Jiri Fronek, Marek Kollar, Marek Petras, Jan Pitha, Rudolf Poledne, Ivana Kralova Lesna

**Affiliations:** ^1^ Laboratory for Atherosclerosis Research, Centre for Experimental Medicine Institute for Clinical and Experimental Medicine Prague Czech Republic; ^2^ Department of Physiology, Faculty of Science Charles University Prague Czech Republic; ^3^ Department of Transplant Surgery Institute for Clinical and Experimental Medicine Prague Czech Republic; ^4^ Department of Pathology Institute for Clinical and Experimental Medicine Prague Czech Republic; ^5^ Department of Epidemiology and Biostatistics, Third Faculty of Medicine Charles University Prague Czech Republic; ^6^ Department of Cardiology Institute for Clinical and Experimental Medicine Prague Czech Republic; ^7^ Department of Anaesthesia and Intensive Medicine, First Faculty of Medicine Charles University and Military University Hospital Prague Czech Republic

**Keywords:** atherosclerotic plaques, comparison, flow cytometry, macrophage polarization, macrophages (subsets), non‐atherosclerotic vessels

## Abstract

The importance of macrophage polarization through atherogenesis is established. However, most studies rely on immunohistological approaches, which have several limitations, such as precluding comprehensive phenotypic analysis. The aim of this study was to perform an alternative analysis of macrophage phenotypes in advanced human atherosclerotic plaques and compare them with their presence in non‐atherosclerotic arteries. Atherosclerotic plaques from 70 individuals indicated for carotid endarterectomy, and samples of non‐atherosclerotic arterial tissue (renal artery, control group) from 45 living kidney donors were processed to obtain immunocytes and incubated with antibodies (CD45, CD14, CD16, CD36, CD163, and CD206) to be analyzed by flow cytometry. Macrophages in the atherosclerotic plaques tend to express CD16 more intensively than in non‐atherosclerotic arterial tissue (transient, CD16^low^ *p* < 0.001, pro‐inflammatory, CD16^high^ *p* < 0.001), and the expression is more closely associated with CD36 expression. Both transient and pro‐inflammatory macrophages are linked with the CD206^−^CD163^+^ or CD206^+^CD163^+^ phenotype in atherosclerotic plaques, while CD206^−^CD163^−^ dominates within the anti‐inflammatory (CD16^neg^) population in the control group. Interestingly, when evaluating all macrophages (regardless of CD16 expression), almost all are CD163^+^ in both groups, supporting the critical importance of using a combination of specific markers. Our results provide a deeper insight into macrophage subpopulations in advanced human atherosclerotic plaques compared with those in non‐atherosclerotic vessels. Additionally, our data highlight the critical importance of using appropriate techniques, such as flow cytometry, allowing for simultaneous analysis of multiple markers to accurately and comprehensively characterize macrophages within the atherosclerotic plaque.

## Graphical abstract

The transition from a non‐atherosclerotic to an atherosclerotic artery is associated with a shift in macrophage phenotypes. In the homeostatic environment of a non‐atherosclerotic artery, macrophages are predominantly anti‐inflammatory, anti‐atherogenic. In contrast, atherosclerotic plaques contain more diverse and pro‐inflammatory macrophage populations, shaped by the inflammatory microenvironment during atherogenesis. Green macrophages represent anti‐inflammatory (CD16^neg^), orange intermediate (CD16^low^) and red pro‐inflammatory (CD16^high^) phenotypes.

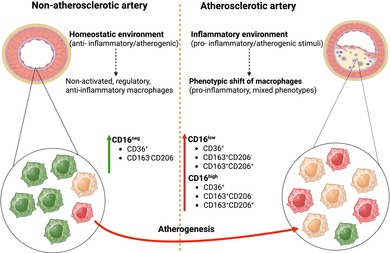

## 1 Introduction

While the activation of multiple cell types within the arterial wall contributes to atherogenesis, macrophages play a pivotal role in driving the inflammatory sterile process. Monocytes migrate into the vessel intima, differentiate into macrophages, and polarize into distinct phenotypes depending on the disease phase [1, 2]. These macrophages can become lipid‐rich foam cells, contributing to plaque buildup, and consequently, thrombogenesis [3, 4]. Besides, macrophages perform efferocytosis and clearing of dead cells, but this process becomes impaired as the disease progresses, leading to necrotic core formation and plaque vulnerability [4, 5]. Macrophages interact with vascular smooth muscle cells, intensifying the inflammatory cycle through the production of additional pro‐inflammatory cytokines and extracellular matrix components, which further promote atherogenesis. These dynamic changes in the atherosclerotic plaque environment drive specific alterations of macrophages to adapt accordingly.

The classical M1/M2 macrophage model was based mainly on in vitro studies and has become outdated. Tissue macrophages bear only a partial resemblance to artificially induced M1 pro‐inflammatory and M2 anti‐inflammatory macrophages. Specifically, human atherosclerotic plaques contain a diverse range of macrophage subtypes [6, 7, 8] including—in addition to M1 and M2 macrophages—anti‐inflammatory M2a [8] and regulatory M2b/c [9]. Other subtypes like M(Hb), Mhem, M4, and TREM2 macrophages contribute variably to plaque stability or inflammation [10, 11, 12].

The relatively well‐described arterial wall‐specific macrophages include M(Hb) stimulated by hemoglobin/haptoglobin and Mhem stimulated by hem [7]. As demonstrated in pre‐clinical research, M(Hb) macrophages exhibit a relatively anti‐inflammatory phenotype [12], as they express the mannose receptor CD206 or CD163 [13, 14]. In vivo in human atherosclerotic plaques, CD163^+^ M(Hb) macrophages prevent their transformation to foam cells [12, 13] via enhanced cholesterol efflux [13] thus potentially slowing the process of atherogenesis. Despite their somewhat anti‐atherogenic properties, hemoglobin oxidation shifts the phenotype toward pro‐inflammatory and rather pro‐atherogenic characteristics [14]. CD163, a hemoglobin scavenger receptor, is primarily expressed by monocytes/macrophages and induced by anti‐inflammatory cytokines. It plays a crucial role in resolving inflammation through its involvement in hemoglobin metabolism and clearance of oxidative stress products. CD163 expression is typically considered an indicator of anti‐inflammatory macrophage polarization [15]. However, its role in the context of atherosclerosis is more comprehensive.

## 2 Aims

The issue of macrophage polarization in the context of atherosclerosis holds significant potential for enhancing our understanding of the risk factors contributing to the inflammatory processes in atherosclerotic plaque. The aim of this study was to identify the major macrophage subpopulations within advanced human atherosclerotic plaques and to provide a robust comparison with those subpopulations in non‐atherosclerotic arteries. Moreover, our study aimed to use flow cytometry to provide a more comprehensive analysis of macrophage phenotypes compared with the widely employed immunohistochemistry techniques with very limited possibility of analyzing cell markers. In our analysis, we applied a marker commonly used to identify blood monocyte subsets for the first time in this context and focused on the co‐expression of additional markers. In line with in vitro studies and our previous results, we examined CD36 linked to specific metabolic polarization and CD163 and CD206, markers involved in immune polarization.

## 3 Material and Methods

### 3.1 Study Participants

Each study participant underwent an endarterectomy, and the removed atherosclerotic plaques were analyzed. A group of living kidney donors with a piece of their renal artery tissue obtained during living kidney donation served as a control group. This part of the most distant renal artery is routinely removed before graft anastomosis.

### 3.2 Processing of the Tissue and Immunocyte Isolation

To process the atherosclerotic plaques, we followed the protocol developed for adipose tissue [16], with modifications specific to this tissue type.

First, atherosclerotic plaques with macroscopically visible calcifications were dissected from the plaques. Next, plaque specimens were minced with scissors and washed with phosphate‐buffered saline (PBS) solution to be subsequently exposed to collagenase IV (2 mg/mL, Sigma‐Aldrich) in 2% bovine serum albumin (BSA)‐PBS solution for 45 min at 37°C. The digested sample was immediately cooled and filtered using two filters (150 and 50 μm), washed twice with cold BSA–PBS solution, and centrifuged (4°C, 300 g, 5 min) to be immediately analyzed by flow cytometry.

The renal artery specimens were obtained from living kidney donors during hand‐assisted retroperitoneoscopic nephrectomy, and their most distant parts (clamped) were processed identically to the atherosclerotic plaque.

### 3.3 Flow Cytometric Analysis

The cells isolated from the atherosclerotic plaques and renal artery tissue, including each participant's blood sample, were incubated with monoclonal antibodies conjugated with specific fluorochromes (CD45‐*Alexa Fluor 405* [AF405] CD14‐*Phycoerythrin* [PE]‐*Cyanin 7* [PC7]; CD16‐*Phycoerythrin‐Texas Red‐X* [ECD]; CD36‐*Fluorescein isothiocyanate* [FITC]; CD163‐PE; CD206‐*Allophycocyanin* [APC]) and *Fixable Viability Dye* (FVD)‐eFluor 780 for 30 min with analysis being performed within 2 h of staining. The CD14, CD16, and CD36 antibodies were purchased from Beckman Coulter (Brea, CA, USA), CD45 from RD system, CD163, CD206 from Biolegend (CA, USA), and FVD from Invitrogen (eBioscience).

**FIGURE 1:**
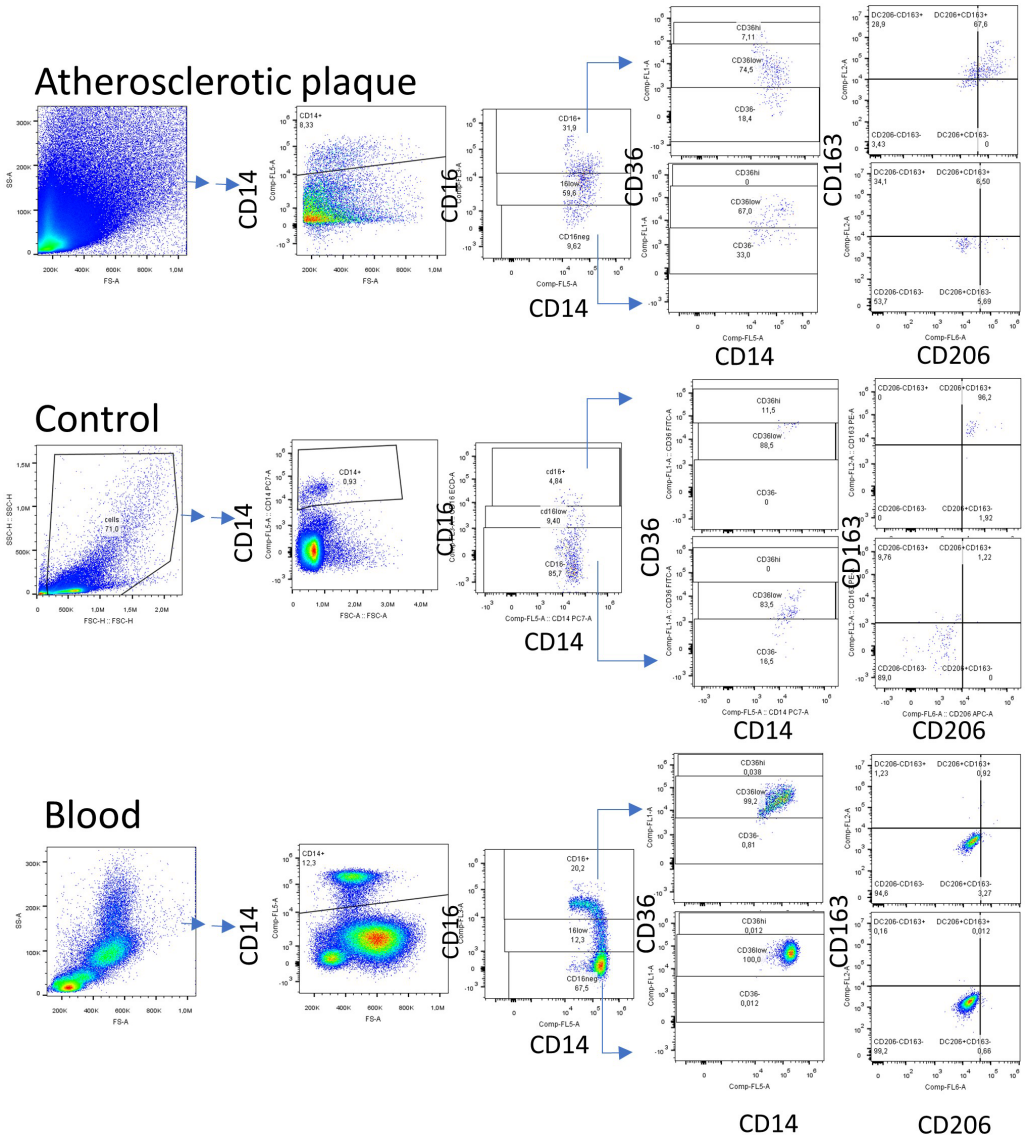
Gating strategy for flow cytometry. CD14^+^ cells were identified from the total cell population following the exclusion of non‐singlets, CD45^−^ cells, and dead cells. The CD14^+^ population was further stratified into CD16^high^, CD16^low^, and CD16^−^ subsets, with gating thresholds determined based on a parallel blood sample analyzed for each subject. Each subset was subsequently classified according to CD36 expression and cross‐gated for CD163 and CD206. The gating parameters were individually established using the corresponding blood sample to ensure consistency and accuracy. All final cell populations expressed at least two phenotypic markers.

All flow cytometric analyses were performed on a Navios Ex3/10 Beckman Coulter flow cytometer (Beckman Coulter, Brea, CA, USA). Only cells expressing CD45 and CD14 and at least one more marker (CD36, CD163, or CD206) were considered macrophages and further analyzed. The gating strategy is shown in Figure 1.

### 3.4 Statistical Analysis

Demographic, clinical, and biochemical variables were reported as proportions or means with standard deviations (SD). Macrophage populations were stated as medians with interquartile ranges (IQR) due to their non‐normal distribution pattern. Categorical variables were assessed using Fisher's exact test. Macrophage populations were analyzed with the unpaired non‐parametric Kruskal–Wallis test followed by Dunn's multiple comparison post hoc test, while other continuous variables were compared using the parametric Student's *t*‐test. All tests were two‐tailed, and the significance level was set at 0.05. Statistical analyses were performed using Prism biostatistics software, version 8 (GraphPad Prism)

## 4 Results

### 4.1 Clinical and Biochemical Characteristics of Study Participants

The atherosclerotic plaques were obtained from 70 subjects, and the non‐atherosclerotic renal arteries (controls) were collected from 45 living kidney donors. There were significant differences in the participants' demographic and clinical characteristics. The atherosclerotic group was expectedly significantly older, with a mean age of 69.3 ± 8.0 years compared with 53.9 ± 10.7 years in the control group (*p* < 0.001). Women were more prevalent in the control group (62%) than in the atherosclerotic group (34%) (*p* < 0.01). Smoking status varied, with 31% of the atherosclerotic group being current smokers and 49% ex‐smokers; in the control group, 24% were current smokers and 20% ex‐smokers (for more than 3 months). The atherosclerotic group had a higher mean BMI (29.3 ± 4.9 kg/m^2^) compared with the control group (25.7 ± 3.4 kg/m^2^, *p* < 0.001). Hypertension was significantly more common in the atherosclerotic group (96%) compared with the control group (22%) (*p* < 0.001), as was dyslipidemia (89% vs. 40%, *p* < 0.001). Subjects were considered dyslipidemic if their fasting plasma cholesterol levels exceeded 5 mmol/L or if they were on lipid‐lowering medication. All lipoprotein parameters were lower in the atherosclerotic group compared with controls, as almost all these patients were treated with lipid‐lowering drugs, as shown in Table 1.

**TABLE 1:** Clinical and biochemical characteristics of individuals with atherosclerotic plaques (*n* = 70) compared to those with non‐atherosclerotic arteries (*n* = 45).

	Atherosclerotic plaques (*n* = 70)	Controls (non‐atherosclerotic arteries; *n* = 45)	*p*
Age	69.3 ± 8.0	53.9 ± 10.7	< 0.001
Women, % (*n*)	34 (24)	62 (28)	< 0.01
Smoker (ex‐smoker), %	31 (49)	24 (29)	< 0.01
Hypertension, % (*n*)	96 (67)	22% (10)	< 0.001
Dyslipidemia, % (*n*)	89 (62)	40 (18)	< 0.001
Statins, % (*n*)	87 (61)	8.9 (4)	< 0.001
Body mass index, kg/m^2^	29.3 ± 4.9	25.7 ± 3.4	< 0.001
Systolic blood pressure, mmHg	145.0 ± 46	128.9 ± 14.8	< 0.001
Diastolic blood pressure, mmHg	83 ± 26	79.5 ± 9.2	0.128
Total cholesterol, mmol/L	3.3 ± 1.5	4.6 ± 0.9	< 0.001
Non‐HDL cholesterol, mmol/L	2.4 ± 1.3	3.3 ± 0.9	< 0.001
LDL‐cholesterol, mmol/L	1.7 ± 1.0	2.9 ± 0.8	< 0.001
Triglycerides, mmol/L	1.3 ± 0.8	1.8 ± 0.9	< 0.05

*Note:* Values are presented as mean *±* standard deviation or percentage. Statistical significance (*p*‐values) indicates differences between the two groups.

### 4.2 Results of the Control Group (Non‐Atherosclerotic Artery)

**FIGURE 2:**
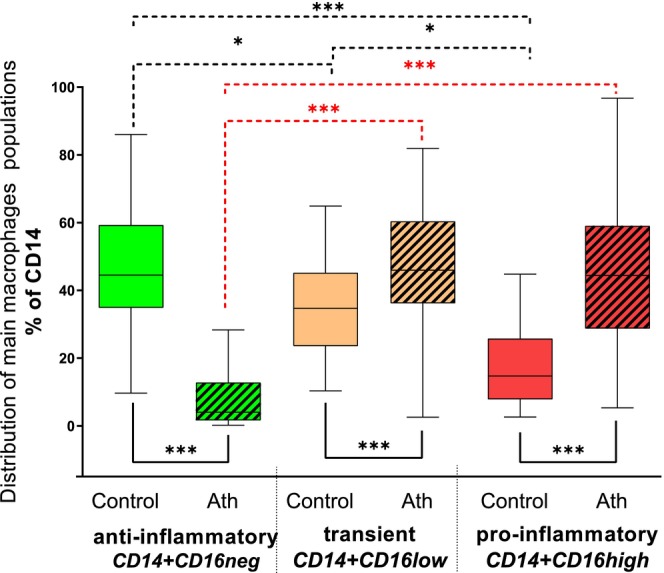
The distribution of main macrophage subpopulations within human atherosclerotic plaques (*n* = 70) and control, non‐atherosclerotic arteries (*n* = 45). Anti‐inflammatory (green boxes, CD14^+^CD16^neg^), transient (orange boxes, CD14^+^CD16^low^), and pro‐inflammatory (red boxes, CD14^+^CD16^high^) macrophages analyzed within human atherosclerotic plaques (Ath, striped boxes, *n* = 70) and in the control group (plain boxes, *n* = 45). Data expressed as the % of CD14^+^ macrophages, presented as median values with interquartile ranges (IQR: Q1–Q3), ***p < 0.001 and *p < 0.05. Dashed lines in the upper part of the graph indicate differences within the macrophage subpopulations in atherosclerotic plaques (red) or control tissues (black), were tested by Wilcoxon tests (paired and nonparametric test), plain lines under the box plots show differences between these groups tested by Mann–Whitney tests (nonparametric test).

Macrophage populations in the control group were classified based on CD16 expression into three main subsets: CD16^−^ (negative; anti‐inflammatory), CD16^low^ (transient), and CD16^high^ (pro‐inflammatory) (Figure 2, plain boxes). Among these, anti‐inflammatory macrophages (CD16^−^) predominated, constituting 44.5% (IQR: 34.9%–59.3%). Transient macrophages (CD16^low^) were less abundant, comprising 34.71% (IQR: 23.6%–45.2%), while pro‐inflammatory macrophages (CD16^high^) represented the smallest fraction at 14.74% (IQR: 7.9%–25.8%).

**FIGURE 3:**
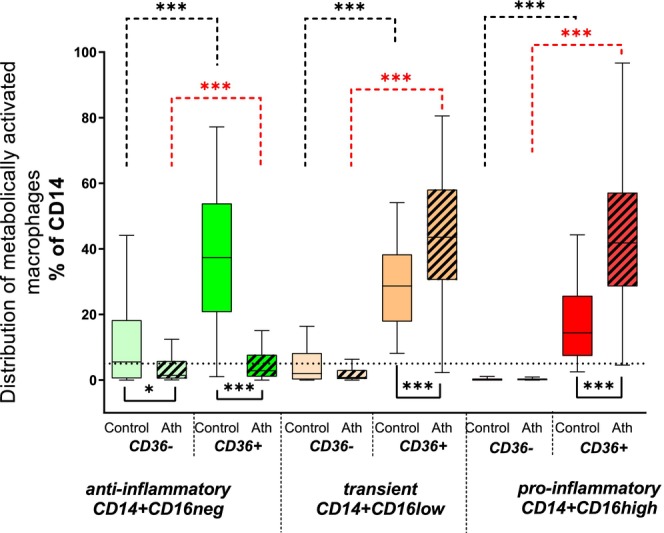
Metabolically activated macrophages within human atherosclerotic plaques (*n* = 70) versus control group (*n* = 45). Macrophages expressing CD36 (metabolic activation marker). Anti‐inflammatory (green boxes, CD14^+^CD16^neg^), transient (orange boxes, CD14^+^CD16^low^), and pro‐inflammatory (red boxes, CD14^+^CD16^high^) macrophages analyzed within human atherosclerotic plaques (Ath, striped boxes, *n* = 70) and in the control group (plain boxes, *n* = 45). Data expressed as the % of CD14^+^ macrophages, presented as median values with interquartile ranges (IQR: Q1–Q3), ***p < 0.001. Dashed lines in the upper part of the graph indicate differences within macrophage subpopulations in atherosclerotic plaques (red) and control tissues (black) tested by Wilcoxon tests (paired and nonparametric test), plain lines under the box plots show differences between these groups tested by Mann–Whitney tests (nonparametric test).

When examining macrophage subpopulations expressing CD36, the most prevalent subset was CD16^−^CD36^+^, accounting for 37.3% (IQR: 20.7%–53.9%). This was followed by CD16^low^CD36^+^ (28.7% [IQR: 17.9%–38.3%]) and CD16^high^CD36^+^ (14.4% [IQR: 7.4%–25.7%]) (Figure 3).

**FIGURE 4:**
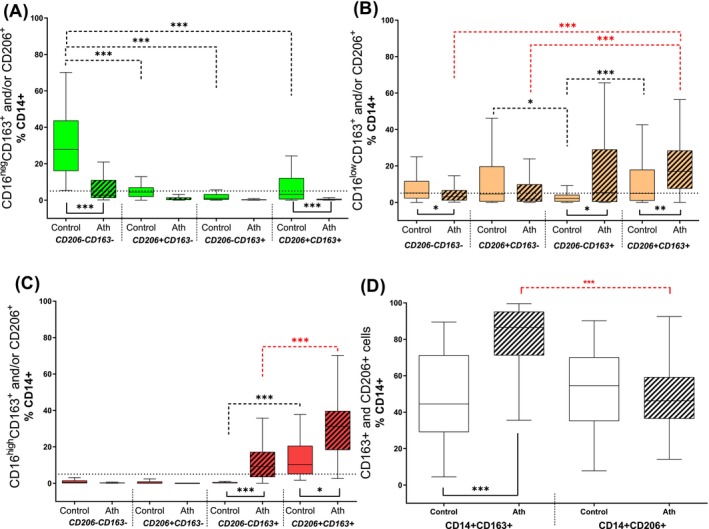
Differences in the distribution of immunologically activated macrophages in the human atherosclerotic plaque (*n* = 70) versus control group (*n* = 45). Macrophages expressing the immunological markers (CD163, CD206). Anti‐inflammatory (green boxes, CD14^+^CD16^neg^), transient (orange boxes, CD14^+^CD16^low^), and pro‐inflammatory (red boxes, CD14^+^CD16^high^) macrophages analyzed within human atherosclerotic plaques (Ath, striped boxes, *n* = 70) and in the control group (plain boxes, *n* = 45). Data expressed as the % of CD14^+^ macrophages, presented as median values with interquartile ranges (IQR: Q1–Q3), ***p < 0.001 and *p < 0.05. Dashed lines in the upper part of the graph indicate differences within the macrophage subpopulations in atherosclerotic plaques (red) and control tissues (black) tested by Wilcoxon tests (paired and nonparametric test), plain lines under the box plots show differences between these groups tested by Mann–Whitney tests (nonparametric test).

To further characterize macrophage diversity, we assessed CD206 and CD163 expression (Figure 4). Within the CD16^−^ macrophage subset, the dominant phenotype was CD206^−^CD163^−^ (27.8% [IQR: 15.9%–43.8%]) (Figure 4A, plain boxes). In contrast, among CD16^low^ macrophages, the frequencies of CD206^+^CD163^−^ and CD206^+^CD163^+^ subpopulations were nearly equal, at 4.6% (IQR: 0.4%–19.8%) and 4.9% (IQR: 0.9%–19.0%), respectively (Figure 4B, plain boxes). Notably, almost all CD16^high^ macrophages (pro‐inflammatory) were double‐positive for CD206 and CD163, representing 10.2% (IQR: 5.0%–20.6%) of total macrophages (Figure 4C, plain boxes).

### 4.3 Main Macrophage Subpopulations in Atherosclerotic Plaques

The distribution of transient (CD16^low^) and pro‐inflammatory (CD16^high^) macrophages within atherosclerotic plaques (Figure 2, striped boxes) was nearly equal, accounting for 46.0% (IQR: 36.2%–60.4%) and 44.4% (IQR: 28.8%–59.1%), respectively. In contrast, anti‐inflammatory macrophages (CD16^−^) were markedly underrepresented, comprising only 4.0% (IQR: 1.6%–12.8%) of the total macrophage population. This significant reduction (*p* < 0.001, compared with transient and pro‐inflammatory macrophages) highlights the rarity of this subtype in atherosclerotic plaques.

Compared with controls, both transient (CD16^low^) and pro‐inflammatory (CD16^high^) macrophages were significantly more abundant in atherosclerotic plaques (*p* < 0.001 and *p* < 0.001, respectively), indicating their enhanced presence in diseased tissue. These findings suggest that the atherosclerotic microenvironment fosters a pronounced polarization of macrophages toward a pro‐inflammatory phenotype.

### 4.4 Subpopulation of Metabolically Activated Macrophages in Atherosclerotic Plaques

Similar to controls, the majority of macrophages in atherosclerotic plaques expressed CD36. Within plaques (Figure 3, striped boxes), CD36 expression was prominent in both transient (CD16^low^) and pro‐inflammatory (CD16^high^) macrophages, with no significant difference between them (43.5% [IQR: 30.0%–58.1%] vs. 41.6% [IQR: 27.9%–56.7%], respectively). Macrophages expressing CD36 were significantly more abundant among CD16^low^ and CD16^high^ subsets compared to the CD16^−^CD36^+^ subpopulation, which constituted only 2.7% (IQR: 0.9%–6.8%) of the total macrophage population (*p* < 0.001).

In contrast to controls, where the CD16^−^CD36^+^ macrophage subpopulation was dominant, its abundance was significantly lower in atherosclerotic plaques (*p* < 0.001). Furthermore, the CD36^+^ transient macrophage subpopulation was more prevalent in atherosclerotic plaques than in controls, and the pro‐inflammatory CD36^+^ macrophage subset was more than twice as abundant in plaques compared to controls.

### 4.5 Macrophage Subpopulations in Atherosclerotic Plaques Based on CD206 and CD163 Expression

To further explore macrophage heterogeneity, we analyzed CD206 and CD163 expression (Figure 4). Among both transient and pro‐inflammatory macrophages, the predominant subpopulation in plaques was CD206^+^CD163^+^, accounting for 17.07% (IQR: 7.5%–18.5%) and 31.2% (IQR: 18.3%–39.7%), respectively (Figure 4B,C, striped orange and red boxes). This was followed by the CD206^−^CD163^+^ subpopulation in atherosclerotic plaque.

Conversely, nearly all anti‐inflammatory macrophages (CD16^−^) were CD206^−^CD163^−^, constituting 2.7% (IQR: 1.3%–11.1%) of the macrophage population (Figure 4A, striped green boxes). Notably, despite lacking CD206 and CD163 expression, these cells exhibited CD36 expression (data not shown).

A primary distinction between plaques and controls was observed in the anti‐inflammatory macrophage populations. In control samples, the CD16^−^ population predominantly lacked CD206 and CD163 expression, whereas in plaques, this subset was significantly diminished (27.8% [IQR: 15.9%–43.4%] in controls vs. 2.7% [IQR: 1.3%–11.1%] in plaques, Figure 4A, green plain and striped boxes).

Regarding transient macrophage subpopulations (Figure 4B), CD206^+^CD163^+^ and CD206^−^CD163^+^ macrophages were significantly more prevalent in atherosclerotic plaques compared to controls (CD206^+^CD163^+^: 17.0% [IQR: 7.52%–28.5%] vs. 4.9% [IQR: 0.9%–18.0%], *p* < 0.01; CD206^−^CD163^+^: 5.3% [IQR: 0.3%–29.1%] vs. 2.2% [IQR: 0.4%–4.1%], *p* < 0.05).

Similarly, when comparing pro‐inflammatory macrophages (Figure 4C), the CD206^+^CD163^+^ subpopulation was significantly more abundant in plaques than in controls (*p* < 0.05; 31.2% [IQR: 18.3%–39.7%] vs. 10.2% [IQR: 5.0%–20.6%]). Additionally, the CD206^−^CD163^+^ subpopulation was markedly more frequent in plaques, whereas it was nearly absent in controls (*p* < 0.001; 1.9% [IQR: 3.4%–17.2%] vs. 0.3% [IQR: 0.0%–0.6%]).

Flow cytometric analysis further revealed distinct CD206 and CD163 expression patterns independent of CD16 expression (Figure 4D). Within atherosclerotic plaques, the majority of macrophages expressed CD163 (86.6% [IQR: 71.2%–95.3%]), whereas CD206^+^ cells comprised approximately half of the macrophage population (46.2% [IQR: 36.4%–59.3%]). Notably, CD163^+^ macrophages were significantly more abundant in plaques compared to controls (*p* < 0.001; 86.6% vs. 44.5% [IQR: 29.1%–71.3%]), while CD206^+^ macrophages were present at similar frequencies in both plaques and controls (54.5% [IQR: 35.1%–70.2%] in controls).

## 5 Discussion

In this study, we used flow cytometry to characterize distinct macrophage subpopulations within the human atherosclerotic plaque to offer new insights into atherogenesis. We identified three main macrophage subtypes based on CD16 expression, noting an increased presence of CD16^high^ macrophages in advanced plaques, which may link these cells to plaque progression. Additionally, plaque macrophages exhibited higher CD36 expression, indicating enhanced metabolic activation compared with non‐atherosclerotic artery macrophages. Interestingly, CD16^high^ macrophages within the plaques co‐expressed also CD206 and/or CD163—markers usually associated with anti‐inflammatory roles—suggesting a complex role in plaque development. Evaluating macrophages regardless of CD16 expression shows that most macrophages are CD163^+^ in both groups. Thus, our data indicate that such evaluating might disguise potential differences and lead to misinterpretation of the results. Unlike histochemistry, flow cytometry is a powerful tool to dissect macrophage heterogeneity in atherosclerosis, providing deeper insight into the immune profile of plaques.

Over the past decade, immune cells have been increasingly recognized as key players in atherogenesis, with macrophages playing a central role. While monocyte polarization is an independent risk factor for atherosclerosis, macrophages within the atherosclerotic plaque are important players at the local level. These cells differentiate in response to signals within the plaque, leading to distinct macrophage subpopulations. Though in vitro studies offer insight into macrophage differentiation, the application of these findings to the arterial wall requires caution. The plaque environment induces unique changes in the macrophages, resulting in hybrid phenotypes that do not strictly match the M1 or M2 profiles seen in vitro [8].

There is a consensus on the phenotypic determination of blood monocytes based on the expression of the CD14 and CD16 markers [17] and this approach has been used in cardiovascular research [18, 19]. CD16 has also been studied in other tissue types such as adipose tissue [20, 21]. Our previous studies have demonstrated its importance in metabolic pro‐inflammatory polarization in adipose tissue macrophages [16, 22]. However, its expression on macrophages within the plaque remains understudied.

We adopted this approach specifically and—in addition to previous studies—distinguished between the CD16^low^ and CD16^high^ macrophages in tissues. Our results indicate a clear association between increased CD16 expression and atherogenesis, with both CD16 positive (either CD16^low^ or CD16^high^) populations making up over 40% of the macrophages in atherosclerotic plaques. Conversely, most of the macrophages in the control group were CD16 negative, making this population five times more prevalent in non‐atherosclerotic tissue. To our knowledge, this is the first study to specifically analyze CD16 expression in macrophages within the human atherosclerotic plaque, and our findings strongly suggest that CD16 expression is indicative of proatherogenic conditions within advanced plaques, consistent with earlier findings in circulating monocytes [17, 23].

We further expanded the characterization of macrophage subpopulations by focusing on additional markers, including CD36, a scavenger receptor implicated in atherogenesis [24, 25]. CD36 is expressed by various cell types (including mononuclear, adipocytes, and endothelial cells) as well as platelets. In macrophages, CD36 plays a key role in binding oxidized low‐density lipoproteins (ox‐LDL) leading to foam cell formation—a critical step in plaque development. In addition, the interaction of CD36 with ox‐LDL is involved in other atherogenic processes and inhibition of macrophage migration [26]. Specific components of internalized LDL directly activate the transcription factor PPARγ, which in turn drives CD36 expression [27]. This creates a positive feedback loop, further increasing the uptake of OxLDL. To date, several studies have investigated CD36 expression in the human atherosclerotic plaque. In an immunohistochemical study, macrophages strongly expressing CD36 were identified as lipid‐laden and localized mainly in the core region [28] but, interestingly, their presence was lower in plaque areas without hemorrhage [7].

Our results show that over 90% of macrophages in atherosclerotic plaques expressed CD36, without a significant difference between the CD16^low^ and CD16^high^ populations. Interestingly, 80% of macrophages in the control group also expressed CD36. However, most of these CD36‐positive macrophages were within the anti‐inflammatory population, constituting the dominant subpopulation, strikingly different compared with plaque macrophages. CD36 within the nonatherosclerotic artery wall might be primarily associated with a distinct macrophage phenotype, possibly linked to tissue homeostasis rather than active lipid uptake and inflammation. The majority of the CD36^+^ anti‐inflammatory macrophages in controls are also CD206^−^ and CD163^−^, which might indicate that this macrophage phenotype might not be fully polarized into a phenotype that only supervises the local environment. Also, microenvironmental factors such as oxidative stress, lipid availability, or cytokine signalingdifferentially regulate CD36 expression in tissue contexts of advanced atherosclerotic plaque and the control artery wall. We must also admit that our model of a nonatherosclerotic arterial wall is oversimplified, as the age of kidney donors implies at least the initial phases of atherogenesis. Our findings align with previous studies on macrophage polarization in adipose tissue, where CD36 high expression was also associated with pro‐inflammatory macrophage subpopulations [16, 29]. However, given the multifaceted role of CD36 in atherogenesis, a more detailed discussion on this topic is beyond the scope of this section and is comprehensively addressed in a recent review [25].

A notable finding in our study was the presence of a small macrophage subpopulation (approximately 10%) negative for both CD16 and CD36, exclusive to non‐atherosclerotic arterial walls. This subpopulation has been linked to areas of plaques lacking hemorrhage in other studies [7]. The clinical significance of these CD36 negative macrophages remains unclear, but their absence in atherosclerotic plaques suggests that they may play a protective role in atherosclerosis‐free tissue.

We also investigated two key macrophage markers associated with inflammation and tissue repair, CD206 and CD163. CD163^+^ macrophages are often found in areas of intraplaque hemorrhage and are involved in the clearance of hemoglobin‐haptoglobin complexes [30]. This process results in the production of iron, which induces oxidative stress and activates further inflammatory pathways. Additionally, CD163^+^ macrophages contribute to the secretion of vascular endothelial growth factor (VEGF) promoting neovascularization within plaques, which may lead to plaque instability and increased risk of rupture [30]. Thus, despite its traditionally anti‐inflammatory association, CD163 expression in atherosclerotic plaques appears to promote proatherogenic mechanisms that can lead to plaque progression and vulnerability.

Our study demonstrated that 80% of macrophages within advanced atherosclerotic plaques expressed CD163 compared with only 40% in controls. This suggests that the plaque microenvironment drives a shift toward a CD163^+^ macrophage phenotype, likely in response to intraplaque hemorrhage and other proatherogenic stimuli. Interestingly, the high proportion of CD163^+^ macrophages in plaques supports previous findings linking this marker to plaque progression and destabilization [30]. It also reinforces the notion that macrophages can adopt a spectrum of functional states depending on the local environment, including phenotypes that are simultaneously involved in both the anti‐inflammatory processes and pathological remodeling.

CD206, known as the mannose receptor, is a marker typically associated with alternatively activated macrophages (M2). CD206 expression is regulated by cytokines such as IL‐4 and IL‐13, and its primary role is related to tissue repair and the clearance of cellular debris, pathogens, and apoptotic cells [31]. Macrophages expressing CD206 exhibit high phagocytic activity, but unlike their CD163 counterparts, they are less involved in the handling of lipids and cholesterol, which are central to foam cell formation [8]. Therefore, CD206^+^ macrophages are thought to play a more reparative role in tissue homeostasis and resolving inflammation.

We observed that a similar proportion of macrophages expressed CD206 in both atherosclerotic plaques and controls, with around 60% of macrophages showing positivity for this marker in both conditions. However, the co‐expression patterns of CD206 with other markers like CD163 differed significantly between the plaques and controls. In non‐atherosclerotic arteries, the majority of macrophages were either double‐positive or double‐negative for both CD163 and CD206, indicating a more uniform polarization toward either a pro‐inflammatory or an anti‐inflammatory state. In contrast, the macrophage population within atherosclerotic plaques was more heterogeneous, with only about 50% of macrophages co‐expressing both CD163 and CD206. This suggests that the plaque microenvironment creates a more complex interplay of pro‐ and anti‐inflammatory signals, leading to a more diverse range of macrophage phenotypes.

Interestingly, our data revealed a substantial increase in transient CD163^+^CD206^−^ macrophages in atherosclerotic plaques, a population that may represent a transitional phenotype influenced by both pro‐inflammatory and reparative cues. These macrophages could be responding to ongoing inflammation and tissue remodeling within the plaque. This hybrid phenotype aligns with the current understanding that macrophages in atherosclerosis do not conform strictly to the classical M1/M2 polarization paradigm. Instead, they adopt a spectrum of phenotypes, reflecting the dynamic and often conflicting signals present in the plaque microenvironment [32].

Overall, the expression patterns of CD163 and CD206 in our study highlight the complexity of macrophage polarization in atherosclerosis. While these markers are traditionally associated with anti‐inflammatory or reparative macrophages, their roles within plaques appear to be more nuanced. CD163, in particular, may drive processes that, while resolving localized inflammation, paradoxically contribute to plaque destabilization through oxidative stress and neovascularization. On the other hand, CD206^+^ macrophages seem to maintain their reparative function but exist alongside other macrophage subpopulations that are actively involved in ongoing inflammation. This balance between reparative and proatherogenic activities reflects the dual roles that macrophages play in both plaque progression and resolution and emphasizes the importance of context in determining macrophage function.

## 6 Limitations

Our study has several limitations, the most important of which is the fact that we do not compare the same tissue, and the control group had a substantially different cardiovascular risk profile. Our controls contained the whole arterial wall whereas the plaque specimens only consisted of parts of the arterial wall. While both carotid and renal arteries are susceptible to atherosclerotic plaque formation, however direct comparative studies analyzing plaque composition between these specific sites are very scarce. The systemic nature of atherosclerosis suggests that androgenetic processes are largely similar across different vascular beds, making significant differences in plaque macrophage characteristics between carotid and renal arteries unlikely. A key advantage of flow cytometry over histological analysis is its ability to simultaneously assess multiple markers at the single‐cell level, allowing for a more detailed characterization of macrophage subpopulations. However, based on our analysis, we can only determine the existence and proportions of these subpopulations, without the ability to compare the total number of macrophages. In contrast, immunohistochemical methods enable the quantification of absolute macrophage numbers but are inherently limited by the number of markers that can be assessed in a single analysis. This limitation underscores the challenges in directly comparing our findings with those obtained through immunohistochemistry, as exemplified by the study of Jinnouchi et al. [7], which demonstrated differences between hemorrhagic and control areas while investigating similar markers of macrophage differentiation. However, results reported to date cannot cover the whole spectrum of macrophage subpopulations. In addition, immunohistochemistry analysis can describe differences between specific areas of the plaque but necessarily fails to draw the whole picture. Importantly, our study focused on the identification and comparison of existing subpopulations rather than on absolute numbers.

## 7 Conclusion

Our results are the first to demonstrate mixed phenotypes of macrophages in the final stages of atherosclerosis. Moreover, to the best of our knowledge, ours is the first study comparing detailed macrophage phenotypes of advanced human atherosclerotic plaques with those found in non‐atherosclerotic human arteries, providing further insight into macrophage polarization within the human atherosclerotic plaque.

## Author Contributions

**Barbora Muffova:** investigation, formal analysis, writing – original draft, visualization; **Sona Kauerova:** methodology, validation, formal analysis, data curation, supervision, writing – review and editing; **Hana Bartuskova:** investigation, validation; **Karel Paukner:** investigation; **Libor Janousek:** methodology, resources; **Jiri Fronek:** resources; **Helena Cermakova:** methodology, investigation; **Marek Petras:** formal analysis, investigation; **Marek Kollar:** conceptualization, methodology; **Jan Pitha:** conceptualization, methodology, writing – review and editing; **Rudolf Poledne:** conceptualization, writing – review and editing; **Ivana Kralova Lesna:** conceptualization, writing – review and editing, supervision, project administration, funding acquisition.

## Disclosure

The authors did not use generative AI or AI‐assisted technologies in the development of this manuscript.

## Ethics Statement

The study was approved by the local Ethics Committee (No. G‐21‐49 and No. G‐16‐06‐22, respectively) according to the Declaration of Helsinki as revised in 2000, and the study was conducted in accordance with the approved protocol.

## Consent

All subjects were thoroughly informed about the study and signed informed consent forms prior to enrollment in the study.

## Conflicts of Interest

The authors declare no conflicts of interest.

## Data Availability

The data are available from the corresponding author on reasonable request.
